# Smoking in the workplace: A study of female call center employees in South Korea

**DOI:** 10.1371/journal.pone.0267685

**Published:** 2022-07-28

**Authors:** Hyunjin Oh, Sunjoo Boo

**Affiliations:** 1 College of Nursing, Gachon University, Incheon, South Korea; 2 College of Nursing·Research Institute of Nursing Science, Ajou University, Suwon, South Korea; University of Jyvaskyla, FINLAND

## Abstract

Smoking among women is characteristically high among call center employees and is associated with various individual and work-related characteristics, which have been paid little attention so far. This study explored the differences in intrapersonal and interpersonal characteristics and environmental factors among Korean women working in call centers by smoking status, based on an ecological model. In this cross-sectional study, an anonymous online survey was conducted among a sample of female employees from three credit card-based call centers (N = 588). Differences in intrapersonal (social nicotine dependence, smoking attitudes, emotional labor), interpersonal (smoking among family or friends, social support), and environmental factors (smoking cessation education, and perceived and preferred smoking policy at work) were compared according to smoking status (smokers, ex-smokers, and never smokers). Approximately 20% (n = 115) were smokers. Smokers were younger, mostly unmarried, had lower education, and had poorer perceived health status than ex- and never smokers. The mean scores for social nicotine dependence and smoking attitude were the highest among smokers, indicating their tendency to underestimate the negative effects of smoking. They also reported the highest level of emotional labor, with about half (50.4%) and almost all (95.7%) reporting smoking behaviors in their families and friends, respectively. Smokers took a lenient stance on the smoking ban policy. The results indicated the necessity to develop tailored smoking cessation programs to motivate female call center employees to quit smoking. As call centers may have a smoking-friendly environment, comprehensive smoking prevention programs considering multilevel factors are required to support smoking cessation.

## Introduction

Globally, smoking is one of the most serious public health threats, killing more than 8 million people annually [1]. Although the total number of smokers in South Korea has been decreasing, the number of female smokers, especially young ones, has increased from 5.5% to 7.5% between 2015 and 2018 [2]. Recent studies have reported that women in call centers have a higher prevalence of smoking, ranging from 20 to 37% [3, 4]. However, owing to the negative cultural and social atmosphere surrounding female smokers in South Korea, these rates may be underreported [5–7]. Despite this negative cultural and social atmosphere, the relatively high rate of smoking among female call center workers may be due to various factors such as work environment, job-related stress, and personal benefits from smoking [3, 4].

Telecalling jobs, commonly perceived as a women’s profession, are characterized by a low wage rate, insecure employment status, and emotional labor in dealing with customer hostility and verbal abuse [8, 9]. Studies have demonstrated that women are more affected by negative psychological consequences of emotional labor, such as burnout and low job satisfaction [10–12]. The generally low socioeconomic status and vulnerable job conditions may affect health behaviors, such as a high prevalence of smoking among female call center employees [9, 13]. Call center work requires employees to control their feelings and reactions to satisfy their customers, even when the customers are hostile and harass them verbally—this is known as emotional labor [3, 4].

**Fig 1:**
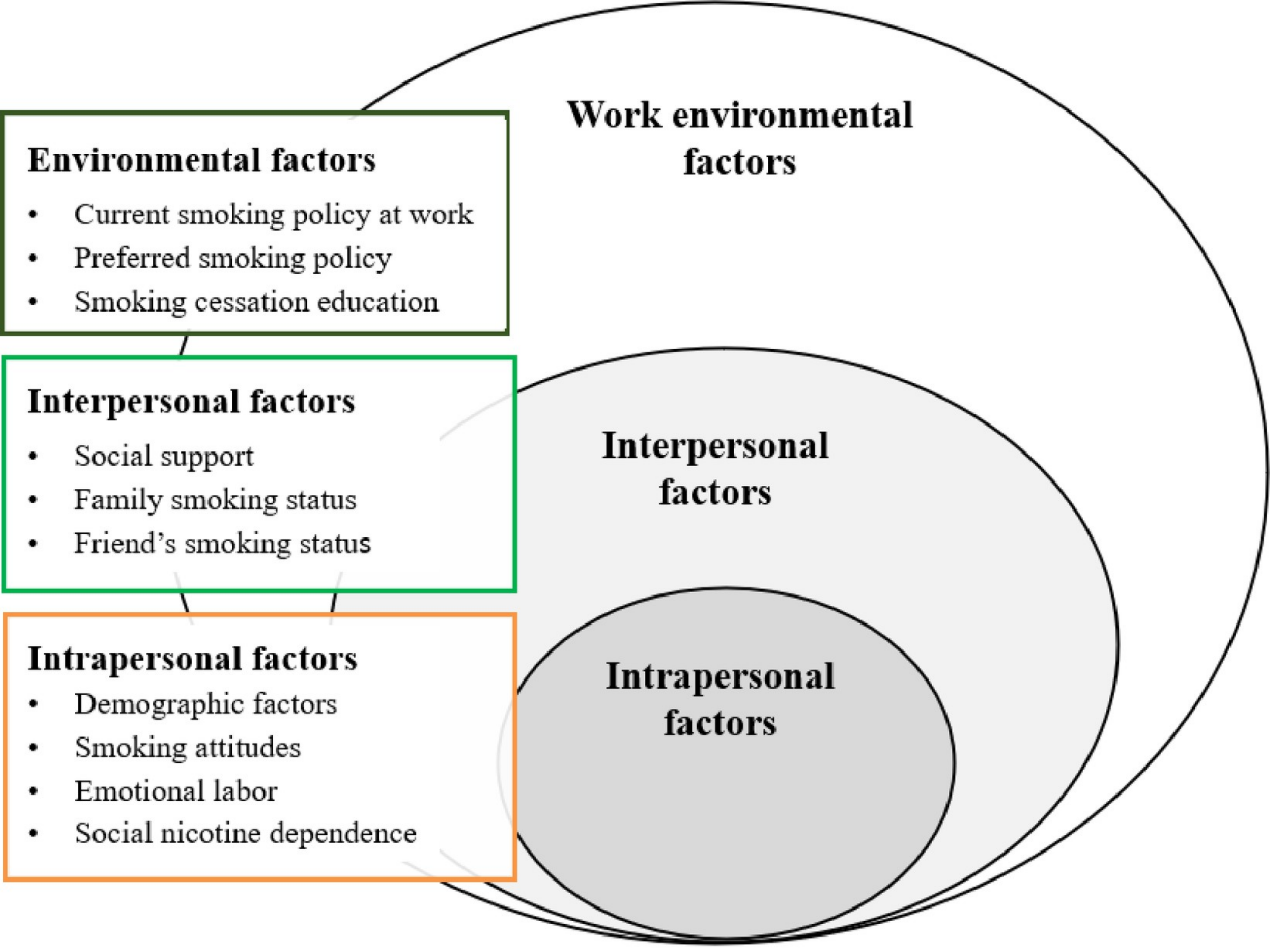
Ecological model for female smoking in call centers.

Smoking behaviors are very complex and comprehensive phenomena which are associated with various personal, social, and environmental factors. Additionally, the ecological model suggests that health-related behaviors are influenced by social and environmental factors [14]—this can be useful for comprehensively understanding behaviors and developing interventions for promoting healthy lifestyles. This study, which aimed to comprehensively understand the factors related to smoking behaviors among female call center employees, was guided by the ecological model (Fig 1). Intrapersonal factors in this model are individual characteristics that influence behavior [14]. In this study, social nicotine dependence, smoking attitudes, and emotional labor were selected to represent intrapersonal factors among female call center employees. Female smokers are more likely to be influenced by non-nicotine factors of smoking [15, 16]. The concept of social nicotine dependency describes the psychosocial linkage with smoking. As pointed out by Kano [17], smokers tend to underestimate the negative effects of smoking and have a positive perception of favorable effects. Smoking attitudes indicate the degree of positive beliefs about smoking [18]. The belief that smoking has harmful health effects may reduce the risk of smoking among women, which is characteristically high in specific occupations [19], and is related to emotional labor, as suggested in previous studies [8, 20].

Interpersonal factors, such as family or friends’ smoking status, may be important risk factors for female smokers, and smoking behavior can spread via such social network members [21]. A previous study demonstrated that smoking cessation among family and close friends was also related to smoking cessation in female call center employees [22]. These studies suggest that family and friends are important influencers of smoking behavior. Studies have also demonstrated that increased levels of social support are associated with reduced health-risk behaviors such as smoking, and emotional support has an overall positive effect on an individual’s health regardless of stressful events [23, 24]. Feeling supported may have a positive emotional buffering effect on call center employees. Although studies have demonstrated the link between social support and smoking status among the general population and patient groups [23, 25, 26], few studies have addressed this relationship in female call center employees as a vulnerable group.

The perception and preference of smoking policy at work were selected as environmental factors for this study. Call centers are described as a “*paradise*” for female smokers in South Korea, who expressed that they could smoke freely and comfortably in the smoking rooms without any social discrimination [3]. A recent study demonstrated that about 16% of smokers started smoking after they started working at a call center [4]. These findings suggest that there may be favorable environmental factors, such as smoking policies at work, that encourage women to smoke. Therefore, it is necessary to investigate the perception of current smoking policies at work and the preferred smoking policy for female call center employees.

It is essential to understand the characteristics of smoking-related factors to identify effective strategies for smoking cessation. Comparing the intrapersonal, interpersonal, and environmental differences between smokers and never smokers within the same occupational group can help identify smoking-related risk factors, which can be crucial in quitting smoking. Although the number of female smokers is steadily increasing in South Korea, little is known about female smoking with respect to ecological models. This study aims to 1) describe the intrapersonal, interpersonal, and environmental factors influencing smoking among female employees in call centers; and 2) explore the differences in those factors based on smoking status.

## Methods

### Study design and participants

A cross-sectional study using an anonymous online survey was conducted from February to April 2021. A priori computation of the sample size using G* Power version 3.1 revealed that 567 participants were required for a three-group plan with an effect size (f) of 0.15, an alpha value of 0.05, and an actual power of 0.90. Potential participants were recruited from three leading South Korean credit card companies’ call centers. Each call center had approximately 1000 employees. The authors contacted unit managers to explain the study’s purpose and procedures, and to distribute the research flyers. The flyers included a cover letter containing the summary of the research as well as a link to the survey which can be completed anonymously. When potential participants clicked on the survey link, they were taken to a webpage that contained detailed descriptions of the research, including data collection procedure and the study’s voluntary and anonymous nature. After reading the detailed information regarding the study and consenting to participate, they were guided to click the research consent button to proceed to the online survey.

The inclusion criteria were female call center employees with at least six months of working experience. Eligibility screening questions were located at the beginning of the survey. Eligibility was determined through self-reports. The online survey was programmed to close automatically for those who did not meet the inclusion criteria. Of the 618 call center employees who completed the initial assessment, 30 did not meet the inclusion criteria yielding a final sample of 588 women. Those who completed the online survey were given a mobile gift voucher worth approximately $10. The study protocol was reviewed and approved by the appropriate ethics committee (AJIRB-SBR-SUR-20-561), and the study was conducted in accordance with the Declaration of Helsinki.

### Measures

#### Smoking status

Smoking status was assessed based on self-reported current smoking status and the number of cigarettes smoked per day. Participants were considered smokers if they reported smoking 100 cigarettes in their lifetime and smoked presently. Ex-smokers had smoked more than 100 cigarettes in their lifetime but did not smoke presently. Never smokers were those who had smoked less than 100 cigarettes in their lifetime and did not smoke presently.

#### Intrapersonal factors

Intrapersonal factors included social nicotine dependence, smoking attitudes, and emotional labor. Social nicotine dependence was assessed using the 10-item Korean version of the Kano Test for Social Nicotine Dependence questionnaire [17, 27]. Each item was scored on a 4-point Likert scale, ranging from 0 (strongly disagree) to 3 (strongly agree). The total scores were calculated by summing the item scores and ranged from 0 to 30. Higher scores indicated a high level of psychosocial dependence on smoking. In this study, Cronbach’s alpha was 0.88.

The 7-item Attitude of Smoking scale was used to measure smoking attitudes. The scale was used for the Teenage Attitudes and Practice Survey by the National Center for Health Statistics in the US and was translated by Lee into Korean [18, 28]. The participants were asked to rate their general perception about smoking and its health effects on a 4-point Likert scale, ranging from 0 (strongly disagree) to 4 (strongly agree). The total smoking attitude score was calculated by averaging the item scores. Higher scores indicated a positive attitude toward smoking. In this study, Cronbach’s alpha was 0.82.

The participants’ level of emotional labor was measured using the Emotional Labor Scale, consisting of 14 items with five subscales: frequency, intensity, variety, surface acting, and deep acting. Surface acting refers to faking and suppressing emotions, while deep acting (DA) implies controlling internal feelings and thoughts [29, 30]. For instance, a surface-acting item was “Pretend to have emotions that I don’t really feel,” while a deep-acting item was “Really try to feel the emotions I have to show as part of my job.” All emotional labor items were measured on a 5-point Likert scale, ranging from 1 (not at all) to 5 (always). Summary scores were calculated by averaging the item scores and ranged from 1 to 5. Higher scores indicated greater emotional labor. This scale was found to have good internal reliability for South Korean emotional laborers [30], and the Cronbach’s alpha in this study was 0.84.

#### Interpersonal factors

In this study, interpersonal factors included family or friends’ smoking status and social support. The smoking behavior of family members who live together was assessed with a yes/no question. Likewise, friends’ smoking behavior was evaluated as a response (yes/no) to whether there were smokers among friends frequently met.

Levels of social support from family, friends, and other significant persons were assessed using the 12-item Korean version of the Multidimensional Scale of Perceived Social Support [31], Each item was scored on a 7-point Likert scale, ranging from 1 (very strongly disagree) to 7 (very strongly agree). The total score was calculated by summing the item scores, with higher scores indicating higher levels of social support. The Cronbach’s alpha was 0.88 at the time of scale development [31] and was 0.96 in this study.

#### Environmental factors

Perception of the current smoking policy at work, smoking policy preferences at work, and smoking cessation education were selected as environmental factors. Perception and preferences for smoking policy at work were self-reported using the questions used in a study by Willemsen et al. [32]. The following question was used to assess the current smoking policy: “How is smoking by employees regulated at your workplace?” The response choices were: (a) Smoking at work is entirely at the discretion of the employees (no explicit policy); (b) there is no ban on smoking except in some general areas that are open to all employees (moderate smoking restriction); (c) smoking is restricted to designated areas (general no-smoking policy); (d) Smoking is not permitted anywhere in our organization (complete smoking ban); (e) do not know. Smoking policy preferences were assessed using a single question with four possible responses: (a) it should be left entirely at the discretion of the employees; (b) a smoking ban should be applied only to public areas, whereas in all other facilities (including the place of work), everyone should be free to smoke; (c) Smoking should be allowed only in designated smoking areas; (d) Smoking should be entirely banned at the workplace. The experience of smoking cessation education was self-reported.

#### Data analysis

The data were analyzed descriptively using IBM SPSS software (version 23.0; IBM Corp., Armonk, NY, USA). Before the analysis, data were inspected for suspected errors, missing data, and outliers, and no issues were identified during the screening. The study variables were summarized as frequencies and percentages for categorical variables and as means (± standard deviations) for continuous variables. Chi-square tests and ANOVAs were used to compare the differences in the study variables according to smoking status. The level of significance was set at *p* < 0.05.

## Results

The distribution of participants’ characteristics and intrapersonal, interpersonal, and environmental factors are summarized in Table 1. About 20% were smokers, 12.1% were ex-smokers, and 68.4% were never smokers. The average age was 41.36 (±8.85) years, and approximately 58% of the participants were married. Approximately 40.1% perceived their health status as good.

**Table 1:** Distribution of participants’ characteristics, intrapersonal, interpersonal, and environmental factors (N = 588).

Characteristics	n (%) or M ± SD
**Participants’ characteristics**	
Age	41.36 ± 8.85
Marital status (married and living together)	340 (57.8)
Educational status (college graduate or above)	351 (59.7)
Monthly household income (≥ 3,000,000 KRW)	338 (57.5)
Perceived health	
Good	236 (40.1)
Fair	261 (44.4)
Poor	91 (15.5)
Smoking status	
Smoker	115 (19.6)
Ex-smoker	71 (12.1)
Never smoker	402 (68.4)
Time working in call centers (months)	65.99 ± 52.96
Number of customers per day	99.98 ± 73.98
Job satisfaction (yes)	346 (58.7)
**Intrapersonal factors**	
Social nicotine dependence	12.38 ± 7.01
Smoking attitude	6.37 ± 4.70
Emotional labor	3.28 ± 0.58
Frequency	3.56 ± 0.78
Intensity	2.80 ± 0.78
Variety	3.39 ± 0.69
Surface acting	3.47 ± 0.71
Deep acting	3.13 ± 0.84
**Interpersonal factors**	
Family smoking status (yes)	242 (41.2)
Friends’ smoking status (yes)	406 (69.0)
Social support	66.81±15.11
**Environmental factors**	
Smoking cessation education (yes)	139 (23.6)
Current smoking policy at work	
No explicit policy	163 (27.7)
Moderate smoking restriction	156 (26.5)
Complete smoking ban	173 (29.4)
Do not know	96 (16.3)
Preferred smoking policy at work	
No explicit policy needed	37 (6.3)
Moderate smoking restriction (smoking ban only applied to public areas)	26 (4.4)
General no smoking policy (smoking only allowed in designated areas)	442 (75.2)
Complete smoking ban	83 (14.1)

For intrapersonal factors, the average levels of social nicotine dependence, smoking attitude, and emotional labor were 12.38 out of 30, 6.37 out of 21, and 3.28 out of 5, respectively. Among the subscales of emotional labor, the mean score for frequency (3.56) was the highest, while the mean score for intensity (2.80) was the lowest. The prevalence of family and friends’ smoking was 41.2% and 69.0%, respectively. Overall, 27.7% of participants perceived no explicit smoking policy at work, while 29.4% reported a complete smoking ban.

Differences in the participants’ characteristics and intrapersonal factors according to smoking status are presented in Table 2. Smokers were younger (*p* < .001), mostly unmarried (*p* < .001), had lower education (*p* = .001), and had poorer perceived health status (*p* < .001) than ex- or never smokers. The mean scores for social nicotine dependence were the highest (20.57) in smokers and lowest in never smokers (9.82), indicating higher levels of psychological and psychosocial dependence on smoking among smokers (*p* < .001), who also experienced higher levels of emotional labor, especially in the subscales of intensity (*p* < .001), variety (*p* < .001), and surface acting (*p* = .001).

**Table 2:** Differences in participants’ characteristics and intrapersonal factors by smoking status (N = 588).

Variables	Never smoker^a^ (n = 402)	Ex-smoker^b^ (n = 71)	Smoker^c^ (n = 115)	x^2^/ F	*p*	Post-hoc test
**Participants’ characteristics**						
Age	43.69±8.57	38.10±8.89	37.36±8.11	32.19	< 0.001	a>b,c
Marital status (married and living together)	263(65.4)	34(47.9)	43(37.4)	32.08	< 0.001	
Educational status (≥ college graduate)	260(64.7)	34(47.9)	57(49.6)	13.17	0.001	
Household income (≥ 3,000,000 KRW/mon)	242(60.2)	41(57.7)	55(47.8)	5.60	0.061	
Perceived health						
Good	189(47.0)	21(29.6)	26(22.6)	32.87	< 0.001	
Fair	168(41.8)	33(46.5)	60(52.2)			
Poor	45(11.2)	17(23.9)	29(25.2)			
Time working in the call center (months)	67.17±52.29	65.56±61.75	62.14±49.79	0.40	0.670	
Number of customers per day	102.40±77.18	93.4558.74	95.61±71.11	0.69	0.503	
Job satisfaction (yes)	252(62.7)	40(56.3)	53(46.1)	10.34	0.006	
**Intrapersonal factors**						
Social nicotine dependence	9.82±5.54	13.66±5.97	20.57±5.60	166.66	< 0.001	a<b<c
Smoking attitude	4.65±3.71	7.46±4.41	11.73±3.62	159.86	< 0.001	a<b<c
Emotional labor	3.21±0.55	3.40±0.51	3.47±0.58	11.801	< 0.001	a<b,c
Frequency	3.52±0.77	3.63±0.77	3.68±0.81	2.05	0.130	
Intensity	2.66±0.76	3.06±0.74	3.13±0.77	21.67	< 0.001	a<b,c
Variety	3.31±0.69	3.54±0.62	3.57±0.69	8.334	< 0.001	a<b,c
Surface acting	3.40±0.68	3.56±0.65	3.67±0.82	7.472	0.001	a<c
Deep acting	3.11±0.81	3.11±0.83	3.20±0.94	0.599	0.550	

**Fig 2:**
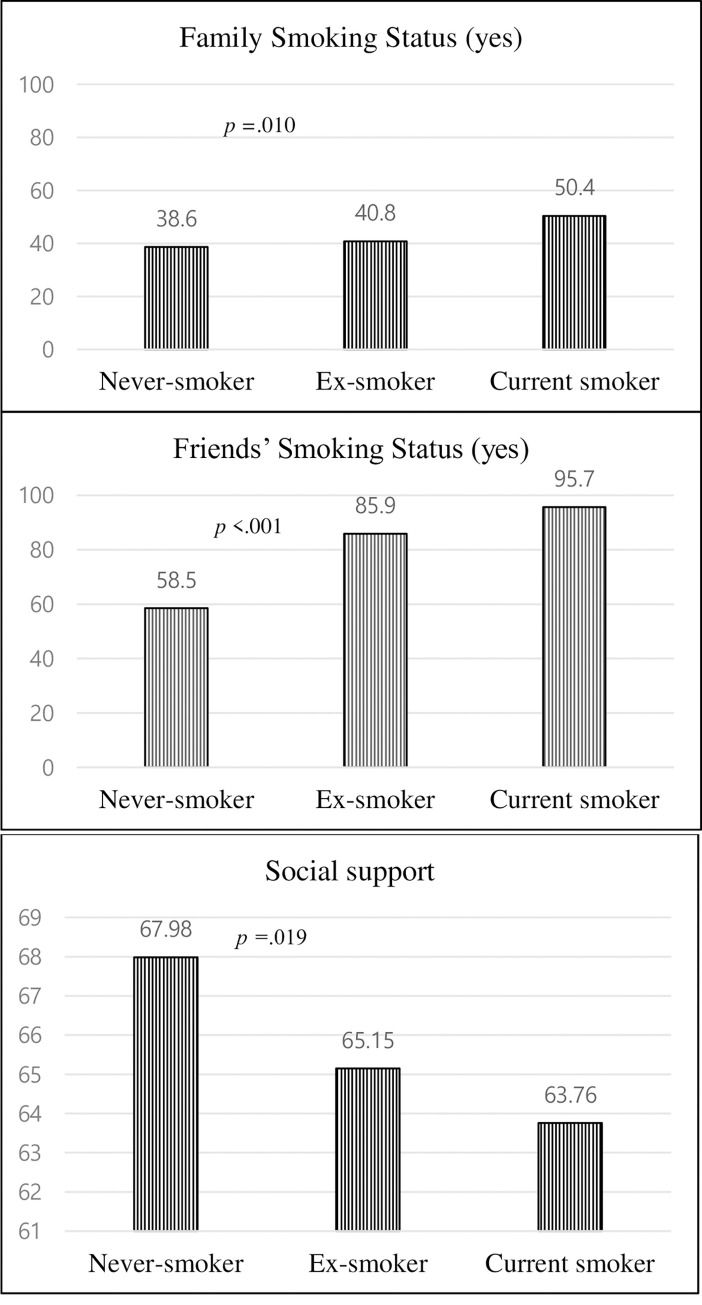
Interpersonal factors by smoking status.

**Fig 3:**
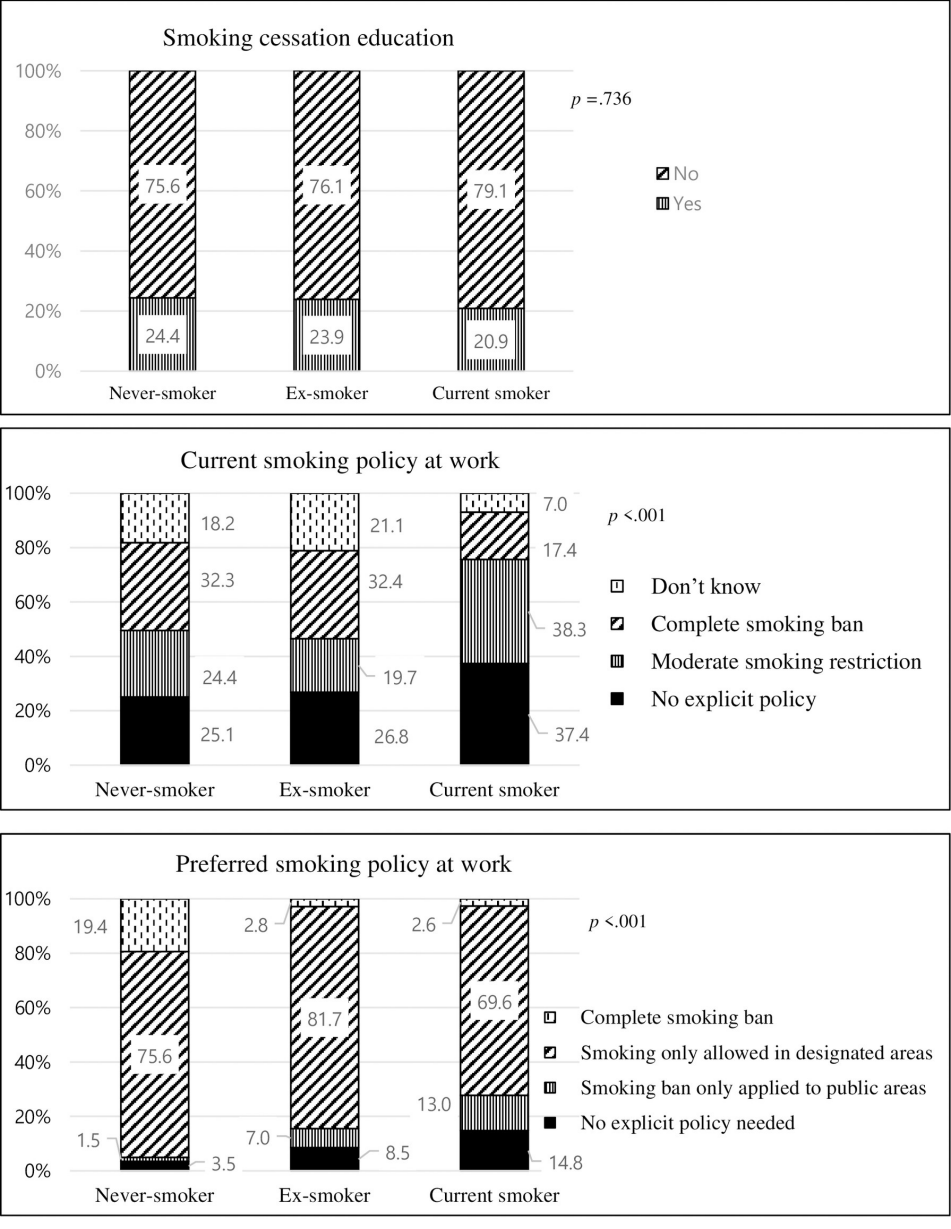
Environmental variables by smoking status.

The distribution of interpersonal and environmental factors by smoking status is graphically depicted separately in Figs 2 and 3. The prevalence of family (*p* = .010) and friends’ smoking (*p* < .001) differed significantly according to the smoking status. It was the highest among smokers, with about half (50.4%) and almost all (95.7%) reported smoking behaviors in their family and friends, respectively (Fig 2). The prevalence of smoking among never smokers’ family and friends were 38.6% and 58.5%, respectively. However, the level of social support was lowest among smokers (63.76) and highest among never smokers (67.98) (*p* = .019). The perceptions and attitudes about smoking policy at work differed according to smoking status (Fig 3). Smokers tended to think that there was no smoking policy at work (37.4%) or that there was a moderate restriction (38.3%) (*p* < .001). Their stance on the smoking ban policy was also lenient: 14.8% of the smokers reported that no explicit policy is needed and 13% preferring smoking ban only applied to public areas. However, 75.6% of never smokers preferred to allow smoking in designated areas, while 19.4% of them preferred a complete smoking ban (*p* < .001). There was no statistical difference in smoking cessation education according to smoking status (*p* = .736).

## Discussion

The purpose of this study was to describe smoking behaviors according to the ecological model and explore the differences in these factors based on the smoking status. Approximately 20% of female call center employees, in this study, were smokers. Considering the social taboo regarding female smoking, the rate might be higher than self-reported rates [33, 34]. In this study, female smokers generally had poor health and lower levels of education, and our finding proves their vulnerable status [33, 35].

Regarding the intrapersonal factors, the levels of social nicotine dependence, smoking attitude, and emotional labor were explored. The average scores of social nicotine dependence among smokers (20.57) were significantly higher than those of ex-smokers (13.66) and never smokers (9.82). Interestingly, the level of social nicotine dependence in this study population was very high compared to the general South Korean population [27] or other ethnic groups [36] in previous study samples. The high social nicotine dependence implies difficulties in quitting smoking [17] and little interest in smoking cessation interventions [37]. Female smokers among call center employees had little intention to quit smoking and seemed to be in a nicotine-dependent culture. In addition, the participants showed a high level of positive beliefs about smoking. Positive smoking attitudes toward smoking were found to be almost three times higher in smokers (11.73) than in never smokers (4.65).

We found that smokers, compared to never smokers and ex-smokers, experienced higher intensity, variety, and surface action in emotional labor. The scores for surface acting were higher in smokers, suggesting a greater tendency to hide or suppress their emotions to satisfy customers [29]. A recent study reported that surface acting is strongly associated with stress responses, and the relationship between emotional labor and occupational stress differed according to smoking status [4]. The results are consistent with previous studies where female employees used smoking as a stress management method [3, 16]. The development of adaptive emotional regulation skills, such as stress management in stress- or anxiety-induced situations, is an essential element of smoking cessation programs for female employees.

Regarding interpersonal factors, the proportion of smokers among family and friends was approximately twice as high for female smokers, referring to a favorable environment and a positive attitude toward smoking. A recent qualitative study on female smoking behavior, attitudes, and experience reported that most female smokers grew up with and were accompanied by their smoker fathers or male friends, thus tending to positively perceive smoking as a social norm and communication tool [38]. Studies have demonstrated that increased levels of social support are associated with reduced health-risk behaviors [39]—this may help female call center employees engage in healthier behaviors. In this study, smokers scored lower than never smokers for social support, consistent with the findings of previous studies [25]. It has been reported that cancer survivors with better mental health and frequent social support are less likely to be smokers [25], implying that abstinence-related social support may be effective in smoking cessation interventions.

Smoking cessation education, perception, and preference regarding the smoking policy at work were explored as environmental factors. The perception and preference regarding the smoking policy at work differed among smokers, ex-smokers, and never smokers. Smokers tended to think that there was no smoking policy at work or that there was a moderate restriction. Their stance on the smoking ban policy was also lenient. According to Kim’s study [3], call centers may provide a favorable environment for female smokers. The study found that a few female call center employees began smoking to fit in their workplace culture. Perceptions and attitudes of female call center employees about smoking policy and their association with smoking rates should be further studied.

For female smokers, call centers may provide a cause and place to smoke simultaneously, which can be considered an obstacle to quitting. Female call center employees go to designated smoking areas, which are completely hidden from outside, to take a break or befriend people while they smoke [3]. Subsequently, efforts to prevent and quit smoking should go beyond individual-level interventions, and workplace-level interventions to identify and manage social factors must be considered. The literature demonstrates that women face various barriers to smoking cessation [40], and there are occupation-specific characteristics of smoking attitudes and behaviors. Therefore, specific strategies considering gender and occupation are needed to help women quit smoking in the future.

While this study revealed some novel findings and supported some previous research results regarding female smoking, two limitations need to be acknowledged to interpret the results appropriately. First, this was a cross-sectional study; therefore, causality could not be implied. Second, smoking status was self-reported and given the social taboo against female smoking in South Korea, it may have been underestimated. For example, one study reported that the prevalence of smoking from the objective measures of smoking and urine cotinine is approximately 5 to 6 times higher than self-reported data [33].

## Conclusions

Smoking-related factors were explored among female call center employees, suggesting the requirement of developing smoking cessation programs based on the needs of female smokers. As the workplace environment can be favorable for smoking, organizational-level smoking cessation-supportive environments and interventions are required while addressing work-related smoking factors.

Thus, it is important to develop comprehensive smoking prevention programs considering these multilevel factors. The literature suggests that individual and work factors should also be considered to meet the individual healthcare needs of female smokers.

## Supporting information

S1 File The data file of 588 female call center employees. (XLSX)
